# Meroterpenoids from Marine Sponge *Hyrtios* sp. and Their Anticancer Activity against Human Colorectal Cancer Cells

**DOI:** 10.3390/md22040183

**Published:** 2024-04-19

**Authors:** Jie Wang, Yue-Lu Yan, Xin-Yi Yu, Jia-Yan Pan, Xin-Lian Liu, Li-Li Hong, Bin Wang

**Affiliations:** 1 Zhejiang Provincial Engineering Technology Research Center of Marine Biomedical Products, School of Food and Pharmacy, Zhejiang Ocean University, Zhoushan 316022, China; yyuelu03@163.com (Y.-L.Y.); 17355410304@163.com (X.-Y.Y.); pjy2388@163.com (J.-Y.P.); lxl19960817@163.com (X.-L.L.); 2 Research Center for Marine Drugs, Department of Pharmacy, Ren Ji Hospital, School of Medicine, Shanghai Jiao Tong University, Shanghai 200127, China

**Keywords:** marine sponge, *Hyrtios* sp., meroterpenoids, invasion, VEGFR-1, colorectal cancer

## Abstract

Two new meroterpenoids, hyrtamide A (**1**) and hyrfarnediol A (**2**), along with two known ones, 3-farnesyl-4-hydroxybenzoic acid methyl ester (**3**) and dictyoceratin C (**4**), were isolated from a South China Sea sponge *Hyrtios* sp. Their structures were elucidated by NMR and MS data. Compounds **2**–**4** exhibited weak cytotoxicity against human colorectal cancer cells (HCT-116), showing IC_50_ values of 41.6, 45.0, and 37.3 μM, respectively. Furthermore, compounds **3** and **4** significantly suppressed the invasion of HCT-116 cells while also downregulating the expression of vascular endothelial growth factor receptor 1 (VEGFR-1) and vimentin proteins, which are key markers associated with angiogenesis and epithelial–mesenchymal transition (EMT). Our findings suggest that compounds **3** and **4** may exert their anti-invasive effects on tumor cells by inhibiting the expression of VEGFR-1 and impeding the process of EMT.

## 1. Introduction

Colorectal cancer (CRC) ranks as the third most prevalent malignancy globally and represents the second leading cause of cancer-related mortality, with an annual incidence of 1.9 million cases and over 0.9 million deaths worldwide [1]. Over 90% of cancer deaths are caused by metastasis of cancer cells [2], making the treatment of metastatic colorectal cancer (mCRC) a significant clinical challenge at present. The surgical prognosis for patients with colorectal cancer is intricately linked to the tumor-node-metastasis (TNM) staging system; patients with stage I–III have five-year survival rates of approximately 90%, 75%, and 50%, respectively. In contrast, those with stage IV disease have a five-year survival rate of less than 5%. Furthermore, a considerable proportion of patients diagnosed with stage II and III colorectal cancer, ranging from 29% to 60%, still experience local recurrence or distant metastases after undergoing surgery [3]. Consequently, addressing tumor metastasis emerges as an urgent imperative in the therapeutic management of colorectal cancer.

Vascular endothelial growth factor receptor (VEGFR), a pivotal regulator of angiogenesis and vascular permeability [4], plays an indispensable role in various biological processes including tumor neovascularization, invasion of tumor cells, and metastasis [5,6]. The VEGFR family primarily comprises three subtypes: namely, VEGFR-1, VEGFR-2, and VEGFR-3 [7]. VEGFR-1, a VEGF receptor with high affinity, exerts specific effects on endothelial cells [8]. In addition to its involvement in tumor vasculature, the activation of VEGFR-1 present in tumor cells through ligand interaction has the potential to stimulate cellular chemotaxis and infiltration into the surrounding extracellular matrix [9]. Colorectal cancer exhibited the presence of VEGFR-1, and its interaction with ligands augmented the migratory and invasive potential of tumor cells [8,9]. Additionally, activation of VEGFR-1 promotes epithelial–mesenchymal transition (EMT), suggesting that VEGFR-1 may initiate EMT to facilitate tumor metastasis [10].

Meroterpenoids are composed of terpenoid units (mevalonic acid pathway) and non-terpenoid units (including polyketide pathways, amino acid pathways, shikimic acid pathways, and other biosynthetic pathways) [11]. The structural and biological diversity of meroterpenoids is attributed to the diverse terpenoid units (including chain length, cyclization, rearrangement, etc.), intricate non-terpenoid moieties, and various modes of molecular aggregation [12,13]. Marine sponges, being a significant reservoir of natural marine products [14], exhibit an annual average production of over 200 novel compounds [15], with a notable abundance in meroterpenoids which display various biological activities, such as anti-tumor [16], anti-inflammation [17], anti-microbial [18], anti-oxidant [19], anti-leishmanial [20] and enzyme inhibitory [21] activities. Meroterpenoids from marine sponges exert anti-tumor effects by arresting the cell cycle progression, inducing apoptosis, suppressing tumor angiogenesis, and inhibiting invasion and metastasis [22,23].

**Figure 1:**
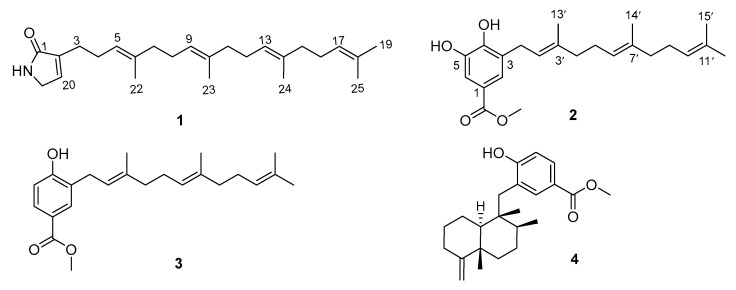
Structures of compounds **1**–**4**.

The marine sponges belonging to the *Hyrtios* genus have demonstrated their potential as a valuable reservoir of novel and biologically active compounds, such as alkaloids, meroterpenoids, and sesterterpenes, and many of them exhibit significant anti-tumor activity [24]. In our ongoing efforts to explore novel and bioactive compounds derived from marine sponges [25,26,27], two new meroterpenoids, hyrtamide A (**1**) and hyrfarnediol A (**2**), as well as two previously reported meroterpenoids, 3-farnesyl-4-hydroxybenzoic acid methyl ester (**3**) [28] and dictyoceratin C (**4**) [29] (Figure 1), were discovered in a marine sponge *Hyrtios* sp. Herein, we discuss the isolation, structural analysis, and cytotoxicity of these meroterpenoids, as well as the anti-invasion effects on colorectal cancer HCT-116 cells of compounds **3** and **4**.

## 2. Results

### 2.1. Structural Determination

**Figure 2:**
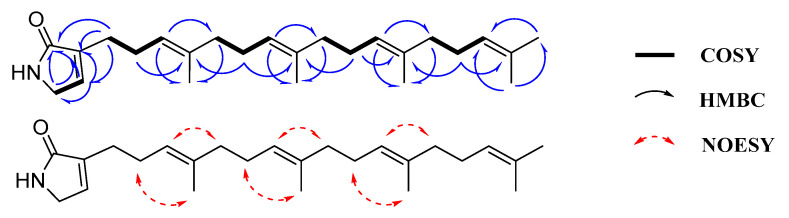
The key COSY, HMBC, and NOESY correlations of compound **1**.

Compound **1** was yielded as a yellowish oil. The molecular formula of **1** was determined as C_25_H_39_NO based on the HRESIMS ion peak at *m*/*z* 392.2914 [M + Na]^+^ (calculated for C_25_H_39_NONa, 392.2929), with seven degrees of unsaturation (Figure S1). The ^1^H NMR data (Table 1, Figure S3) showed five methyls at *δ*_H_ 1.60 (br s, H-22, 23, 24, 25) and 1.68 (s, H-19), eight methylenes at *δ*_H_ 1.98 (m, H-7, 11, 15), 2.06 (m, H-8, 12, 16), 2.26 (q, *J* = 7.0 Hz, H-4), and 2.33 (t, *J* = 7.0 Hz, H-3), one *N*-methylene at *δ*_H_ 3.91 (t, *J* = 1.5 Hz, H-21), and five olefinic protons at *δ*_H_ 6.73 (q, *J* = 1.5 Hz, H-20), 5.15 (t, *J* = 7.0 Hz, H-5) and 5.11 (m, H-9, 13, 17). The ^13^C NMR and DEPT135 spectra (Table 1, Figure S4) exhibited 25 carbon signals, including five methyl carbons [*δ*_C_ 16.2 (C-23, 24), 16.3 (C-22), 17.9 (C-25), 25.9 (C-19)], eight methylene carbons [*δ*_C_ 25.8 (C-3), 26.1 (C-4), 26.9 (C-12, 16), 27.0 (C-8), 39.9 (C-7, 11, 15)], one *N*-methylene carbon *δ*_C_ 46.6 (C-21), five methine carbons [*δ*_C_ 124.6 (C-17), 124.4 (C-9, 13), 123.5 (C-5), 137.7 (C-20)], five quaternary carbons [*δ*_C_ 131.5 (C-18), 135.1 (C-14), 135.2 (C-10), 136.3 (C-6), 139.7 (C-2)], and one carbonyl carbon *δ*_C_ 175.3 (C-1). The COSY correlation of H-20/H-21, combined with HMBC correlations from H-20 (*δ*_H_ 6.73) to C-1 (*δ*_C_ 175.3), C-2 (δ_C_ 139.7), and C-21 (*δ*_C_ 46.6), from H-21 (*δ*_H_ 3.91) to C-1 (*δ*_C_ 175.3) and C-2 (*δ*_C_ 139.7), and from H-3 (*δ*_H_ 2.33) to C-1 (*δ*_C_ 175.3), C-2 (*δ*_C_ 139.7), and C-20 (*δ*_C_ 137.7), led to the identification of a 3-en-α-pyrrolidone fragment (Figure 2, Figures S6 and S7). The geranylgeranyl fragment was determined by COSY correlations of H-3/H-4/H-5, H-7/H-8/H-9, H-11/H-12/H-13, and H-15/H-16/H-17 and HMBC correlations from H-3 (*δ*_H_ 2.33) to C-5 (*δ*_C_ 123.5), from H-4 (*δ*_H_ 2.26) to C-2 (*δ*_C_ 139.7), C-5 (*δ*_C_ 123.5), and C-6 (*δ*_C_ 136.3), from H-5 (*δ*_H_ 5.15) to C-7 (*δ*_C_ 39.9) and C-22 (*δ*_C_ 16.3), from H-7, 11, 15 (*δ*_H_ 1.98) to C-5 (*δ*_C_ 123.5), C-9, 13 (*δ*_C_ 124.4), C-17 (*δ*_C_ 124.6), C-22 (*δ*_C_ 16.3) and C-23, 24 (*δ*_C_ 16.2), from H-9, 13, 17 (*δ*_H_ 5.11) to C-7, 11, 15 (*δ*_C_ 39.9), C-19 (*δ*_C_ 25.9) and C-23, 24 (*δ*_C_ 16.2), from H-19 (*δ*_H_ 1.68) to C-25 (*δ*_C_ 17.9), C-17 (*δ*_C_ 124.6) and C-18 (*δ*_C_ 131.5), and the methylene carbon C-3 was located at C-2 on the 3-en-α-pyrrolidone fragment which was demonstrated by the HMBC correlations from H-3 (*δ*_H_ 2.33) to C-1 (*δ*_C_ 175.3), C-2 (*δ*_C_ 139.7), and C-20 (*δ*_C_ 137.7), and the chemical shift of C-2 (*δ*_C_ 139.7, quaternary carbon). Therefore, the planar structure of compound **1** was obtained. The NOESY correlations (Figure S8) of H-4/H-22, H-5/H-7, H-8/H-23, H-9/H-11, H-12/H-24, and H-13/H-15, and the shielded chemical shifts of C-22 (*δ*_C_ 16.3), C-23 (*δ*_C_ 16.2), and C-24 (*δ*_C_ 16.2) suggested that Δ^5,6^, Δ^9,10^, and Δ^13,14^ double bonds were both *E*. Compound **1** was named hyrtamide A.

**Figure 3:**
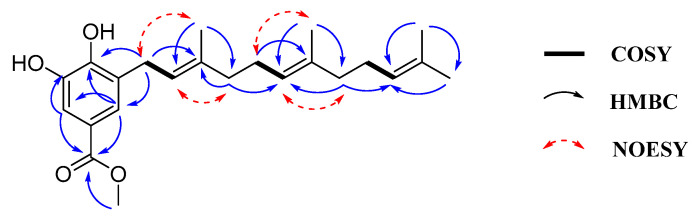
The key COSY, HMBC, and NOESY correlations of compound **2**.

Compound **2** was a yellowish powder, and exhibited a positive HRESIMS ion peak at *m*/*z* 373.2377 [M + H]^+^ (calculated for C_23_H_33_O_4_, 373.2379), indicating its molecular formula as C_23_H_32_O_4_, corresponding to eight degrees of unsaturation (Figure S9). The ^1^H and ^13^C NMR data (Table 1, Figures S11 and S12) were characterized by the presence of two aromatic methines at *δ*_H_ 7.46 (br s, H-6)/*δ*_C_ 114.7 and *δ*_H_ 7.44 (br s, H-2)/*δ*_C_ 124.0, suggesting the two aromatic protons were in the meta position. Further examination of the ^1^H and ^13^C NMR data of **2** revealed the presence of three olefinic methines [*δ*_H_ 5.10 (m, H-6′, 10′) and 5.34 (m, H-2′); *δ*_C_ 123.8, 124.5, and 121.3], an oxygenated methyl [*δ*_H_ 3.87 (s), *δ*_C_ 52.2], and four methyls [*δ*_H_ 1.60 (br s, H-14′, 15′), 1.58 (s, H-12′), and 1.79 (s, H-13′); *δ*_C_ 16.3, 17.9, 25.9, and 16.5]. The HMBC correlations (Figure 3, Figure S14) from H-2 (*δ*_H_ 7.44) to 1-C=O (*δ*_C_ 167.4), C-4 (*δ*_C_ 147.0), and C-6 (*δ*_C_ 114.7), from H-6 (*δ*_H_ 7.46) to 1-C=O (*δ*_C_ 167.4) and C-5 (*δ*_C_ 146.7), and from -OCH_3_ (*δ*_H_ 3.87) to 1-C=O (*δ*_C_ 167.4) indicated the presence of a 4,5-dihydroxybenzoic acid methyl ester. The COSY correlations (Figure S15) of H-1′/H-2′, H-5′/H-6′, and H-9′/H-10′, combined with HMBC correlations from H-1′ (*δ*_H_ 3.40) to C-3′ (*δ*_C_ 139.3), from H-4′ (*δ*_H_ 2.11, 1.97) to C-3′ (*δ*_C_ 139.3) and C-6′ (*δ*_C_ 123.8), from H-8′ (*δ*_H_ 2.11, 1.97) to C-6′ (*δ*_C_ 123.8) and C-9′ (*δ*_C_ 26.6), from H-15′ (*δ*_H_ 1.60) to C-10′ (*δ*_C_ 124.5) and C-12′ (*δ*_C_ 25.9), from H-14′ (*δ*_H_ 1.60) to C-6′ (*δ*_C_ 123.8) and C-8′ (*δ*_C_ 39.9), from H-13′ (*δ*_H_ 1.79) to C-2′ (*δ*_C_ 121.3) and C-4′ (*δ*_C_ 39.9) indicated the presence of a farnesyl fragment. The HMBC correlations from H-1′ (*δ*_H_ 3.40) to C-2 (*δ*_C_ 124.0), C-3 (*δ*_C_ 127.3), and C-4 (*δ*_C_ 147.0) revealed that the farnesyl moiety was attached to C-3 of the benzene ring. E-configurations of the Δ^2′,3′^ and Δ^6′,7′^ double bonds in the farnesyl fragment of **2** were confirmed based on the NOESY interactions (Figure S16) of H-2′/H-4′, H-6′/H-8′, H-1′/H-13′, and H-5′/H-14′, and the shielded chemical shifts of C-13′ (*δ*_C_ 16.5) and C-14′ (*δ*_C_ 16.3). Consequently, the structure of **2** was assigned as 3-farnesyl-4,5-dihydroxybenzoic acid methyl ester and named hyrfarnediol A.

The two known meroterpenoids, 3-farnesyl-4-hydroxybenzoic acid methyl ester (**3**) [28] and dictyoceratin C (**4**) [29], were identified by comparing their NMR and MS data (Figures S17–S22) with the values reported in the existing literature.

### 2.2. Bioactive Assay

#### 2.2.1. Cytotoxicity of Compounds **1**–**4** against HCT-116 Cells

Colorectal cancer stands as one of the most frequently diagnosed malignant neoplasms within the gastrointestinal tract. The World Health Organization’s statistics place CRC as the third most common type of cancer globally, characterized by its high incidence and mortality rates, along with a significant propensity for metastasis [30]. Numerous meroterpenoids have been identified to exhibit potent inhibitory effects on HCT-116 cells—a cell line derived from human colorectal carcinoma [23]. In view of this, we assessed the cytotoxic effect against HCT-116 cells of compounds **1**–**4** utilizing the MTT assay method (Table 2). Compounds **2**–**4** showed weak cytotoxicity towards HCT-116 cells, with IC_50_ values of 41.6, 45.0, and 37.3 μM, respectively; however, compound **1** failed to manifest any inhibitory effect even at a concentration level of up to 54 μM. Nevertheless, owing to the insufficient quantity of compound **2** for subsequent activity analyses, we opted to proceed with cell invasion experiments using compounds **3** and **4**. 

#### 2.2.2. Anti-Invasive Activity of **3** and **4** in HCT-116 Cells

**Figure 4:**
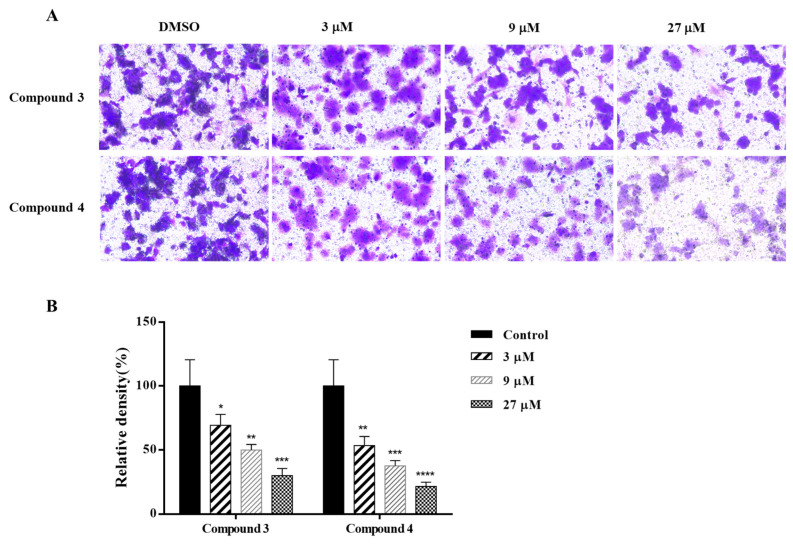
The inhibiting effects of compounds **3** and **4** on the invasion activity of HCT 116 cells. (**A**) Representative images in the invasion assay; (**B**) Relative percentage of invaded cells. Data are presented as the mean ± standard deviation (*n* = 3). * *p* < 0.05, ** *p* < 0.01, *** *p* < 0.001, **** *p* < 0.0001.

More than 90% of cancer deaths result from the metastasis of cancer cells [2]. Invasion takes place at an early stage of the metastatic process and represents a pivotal step in this progression [31,32]. Many meroterpenoids demonstrated significant anti-invasive activity, including sponge-derived terpene quinones/phenols strongylophorine-26 and avinosol [33,34]. To evaluate the impact of compounds **3** and **4** on HCT-116 cell invasiveness, we employed Transwell assays. The results demonstrated that at concentrations of 3, 9, and 27 μM, there were notable decreases in cell invasion capabilities in a dose-dependent manner (Figure 4).

#### 2.2.3. Effects of **3** and **4** on Expressions of VEGFR-1 and Vimentin

To elucidate the underlying mechanisms responsible for the inhibitory effects of compounds **3** and **4** on cell invasion in HCT-116 cells, proteins associated with EMT and metastasis were analyzed. VEGFR-1 is one of the VEGF receptors, which in tumor cells can be activated by its ligands (such as VEGF-B and PIGF) to significantly increase the invasion capabilities of the tumor cells [9]. This receptor is also closely associated with the process of EMT, which is linked to metastasis [10]. Tumor cells undergoing EMT exhibit alterations in relevant molecular biomarkers [35]. Vimentin serves as a critical biomarker for EMT, which is typically expressed in mesenchymal cells. Its expression levels become upregulated during the process of cancer metastasis [36]. During EMT, vimentin undergoes reorganization and mediates signaling pathways, all the while providing structural support to various cellular organelles due to its unique viscoelastic properties [36]. Moreover, it facilitates cell migration through the formation of cellular protrusions, reduction in cell adhesion, and enhancement of migratory capacity [36]. Additionally, vimentin modulates DNA repair pathways to facilitate EMT and confer cellular resilience against diverse stresses encountered during the process of cancer invasion [36]. Overall, vimentin appears to play a crucial role in mediating metastasis through EMT processes [36].

**Figure 5:**
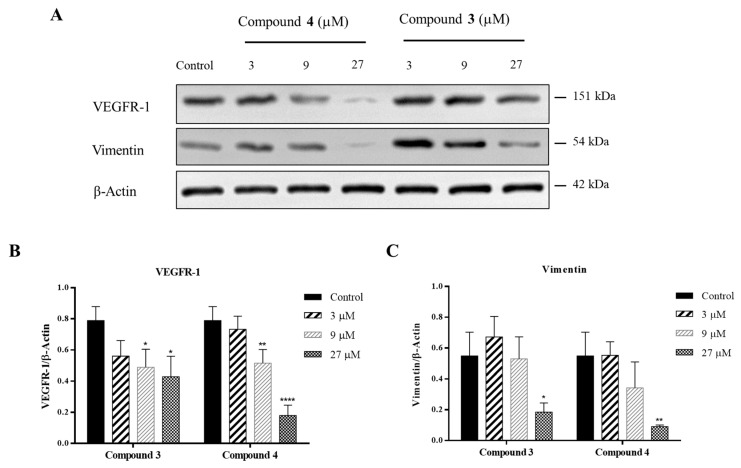
The effects of compounds **3** and **4** on the protein levels of VEGFR-1 and vimentin in HCT 116 cells. (**A**) Western blot analysis of VEGFR-1 and vimentin, β-actin was used as the internal control; (**B**) Relative protein level of VEGFR-1 against β-actin; (**C**) Relative protein level of vimentin against β-actin. Data are presented as the mean ± standard deviation (*n* = 3). * *p* < 0.05, ** *p* < 0.01, **** *p* < 0.0001.

In this study, the effects of compounds **3** and **4** on the expression of migration-related proteins VEGFR-1 and vimentin in HCT-116 cells were examined using Western blot analysis at concentrations of 3, 9, and 27 μM (Figure 5). Experimental results indicated that compounds **3** and **4** significantly downregulated the expression of VEGFR-1 and vimentin in a concentration-dependent manner, indicating that compounds **3** and **4** may inhibit the expression of VEGFR-1, thereby impeding the EMT process. 

## 3. Discussion

A meroditerpenoid, hyrtamide A (**1**), and three sesquiterpene phenols, hyrfarnediol A (**2**), 3-farnesyl-4-hydroxybenzoic acid methyl ester (**3**) and dictyoceratin C (**4**) were isolated from a marine sponge *Hyrtios* sp. In the cytotoxicity assay, compounds **2**–**4** demonstrated weak cytotoxic activity, while no activity was observed for hyrtamide A (**1**), which suggests that the presence of the phenol fragment in compounds **2**–**4** may play a vital role in cytotoxicity. Compounds **3** and **4** exhibited anti-invasive effects, in contrast to previously reported sesquiterpene phenols/quinones, such as avarol, avarone, and ilimaquinone which did not show any anti-invasive activity [33,34]. Through structural comparison, we hypothesize that the phenolic hydroxyl and methyl ester groups present in compounds **3** and **4** may serve as the active functional groups responsible for their anti-invasive activity.

Compounds **3** and **4** downregulated the expression of VEGFR-1 and vimentin proteins. Several reported phenols and sesquiterpene quinones inhibited VEGFR expression [37,38,39,40]. We consider that hydroxyl groups play an important role in inhibiting VEGFR expression. Additionally, in compounds **3** and **4**, the methyl ester group may also play an indispensable role. Most naturally derived VEGFR inhibitors primarily target VEGFR-2 and VEGFR-3; however, there are only few compounds known to inhibit VEGFR-1 [37,38,39,40]. VEGFR-1 is closely related to angiogenesis promotion along with evidence suggesting its involvement in inducing EMT to facilitate tumor cell invasion [10]. Compounds **3** and **4** inhibited the expression of VEGFR-1 protein and suppressed vimentin simultaneously. Thus, we propose that compounds **3** and **4** likely inhibit EMT by suppressing the expression of VEGFR-1, thereby restraining HCT-116 cells invasion.

## 4. Materials and Methods

### 4.1. General Experimental Procedures

The NMR experiments were conducted on Bruker Avance DRX-600 and Bruker AMX-400 MHz NMR spectrometers (Bruker BioSpin, Bremen, Germany) in CDCl_3_ (*δ*_H_ 7.26, *δ*_C_ 77.16). HRESIMS spectra were recorded on an Agilent 6230 TOF mass spectrometer (Agilent Technologies, Santa Clara, CA, USA). MPLC was carried out on an Interchim PuriFlash 450 instrument (Interchim, Montlucon, France). TLC was carried out on silica gel HSGF_254_ plates (Yantai Jiangyou Silica Gel Limited Company, Yantai, China). Column chromatography was conducted using Sephadex LH-20 (18−110 μm, Pharmacia Co., London, UK) and ODS C_18_ (15 μm, Santai Technologies, Inc., Changzhou, China). HPLC was performed using a Waters 1525 equipped with a Waters 2998 PDA detector (Waters, Milford, CT, USA). A C_18_ column (YMC-Pack Pro, 250 × 10 mm, 5 μm, YMC, Kyoto, Japan) was used for RP HPLC.

### 4.2. Sponge Material

The marine sponge sample was collected from the vicinity of Yongxing Island in the South China Sea in May 2013. The species was previously identified and described as a *Hyrtios* sp. [29]. A voucher specimen (no. 1312) is deposited at Research Center for Marine Drugs, Department of Pharmacy, Ren Ji Hospital, School of Medicine, Shanghai Jiao Tong University.

### 4.3. Extraction and Isolation

The sponge *Hyrtios* sp. (0.2 kg, dry weight) was subjected to a triple extraction with 95% EtOH to produce an ethanolic extract. This extract underwent partitioning between petroleum ether and a 90% methanol–water mixture at equal ratios. The methanol–water fraction was then diluted to 60% with water and further partitioned using CH_2_Cl_2_, resulting in a CH_2_Cl_2_ extract weighing 1.3 g. The CH_2_Cl_2_ extract was separated using a Sephadex LH-20 column with CH_2_Cl_2_/MeOH (1:1), resulting in the isolation of three fractions (A–C). Fraction B (0.86 g) was subjected to MPLC (MeOH/H_2_O, 50–100%) and yielded six fractions (B1–B6). 

Purification of subfraction B4 via RP HPLC (MeOH/H_2_O, 80%) afforded two compounds: dictyoceratin C (**4**, *t*_R_ = 85 min, 29.0 mg) and 3-farnesyl-4-hydroxybenzoic acid methyl ester (**3**, *t*_R_ = 102 min, 13.3 mg). Similarly, subfraction B5 when processed through RP HPLC (MeCN/H_2_O, 80%) produced hyrfarnediol A (**2**, *t*_R_ = 48 min, 2.6 mg). Hyrtamide A (**1**, *t*_R_ = 70 min, 3.0 mg) was purified from subfraction B6 utilizing RP HPLC (MeCN/H_2_O, 85%). 

### 4.4. Compound Characteristics

Hyrtamide A (**1**): Yellowish oil; UV (DAD from MeOH/H_2_O) *λ*_max_ 221 nm; ^1^H and ^13^C NMR (600/150 MHz, CDCl_3_) data, as shown in Table 1; HRESIMS *m*/*z* 392.2914 [M + Na]^+^ (calcd. for C_25_H_39_NONa, 392.2929).

Hyrfarnediol A (**2**): Yellowish powder; UV (DAD from MeOH/H_2_O) *λ*_max_ 199 and 268 nm; ^1^H and ^13^C NMR (400/100 MHz, CDCl_3_) data, as shown in Table 1; HRESIMS *m*/*z* 373.2377 [M + H]^+^ (calcd. for C_23_H_33_O_4_, 373.2379).

### 4.5. Cytotoxicity Test

The HCT-116 cell line, sourced from Shanghai Institute of Biochemistry and Cell Biology, Chinese Academy of Sciences (Shanghai, China), was cultured at 37 °C with 5% CO_2_ in McCoy’s 5A medium (Procell, Wuhan, China). The HCT-116 cells were seeded at a concentration of 1 × 10^4^ cells/well and incubated for 24 h. The cell line was treated with test compounds (at concentrations of 10.0, 20.0, 40.0, and 80.0 μM) and the positive control (doxorubicin, at concentrations of 1.0, 2.0, 4.0, and 8.0 μM) for 48 h. Then, they were co-incubated with MTT solution (20 μL, Sinopharm Chemical Reagent Co., Ltd., Shanghai, China) at 37 °C for 4 h. The absorbance was quantified at a wavelength of 490 nm using a microplate reader (Synergy, BioTek Instruments, Inc., Winooski, VT, USA). The cytotoxicity assays were performed with at least three replicates [41].

### 4.6. Invasion Assay

The HCT-116 cells were harvested and suspended in a medium without serum at a concentration of approximately 1 × 10^6^ cells/mL. The upper compartment of a transwell chamber, pre-coated with Matrigel (BD Biosciences, Franklin Lakes, NJ, USA), was then seeded with these cells. The lower chamber was filled with a medium containing 20% FBS (Genom, Hangzhou, China) and various concentrations of compounds **3** and **4** (with DMSO as the control). The cells were treated with 4% paraformaldehyde (Biosharp, Hefei, China) and fixed for 24 h. The non-invasive cells located on the outermost layer of the membrane were removed lightly using a sterile cotton swab. The membrane-bound cells were subjected to crystal violet staining for 10 min, followed by three rounds of washing with PBS (Genom, Hangzhou, China), and the captured images were observed under an inverted microscope (Olympus, Tokyo, Japan).

### 4.7. Western Blot

After treating HCT-116 cells with DMSO or different concentrations of compounds **3** and **4** for 24 h, the cells were rinsed with PBS that had been pre-cooled, followed by lysis using 150 μL of lysis buffer. The samples were incubated on ice for 30 min and vigorously shaken to ensure complete cell lysis. Subsequently, centrifugation was performed at 12,000 rpm and 4 °C for 15 min. The supernatant was collected for protein concentration analysis. SDS-PAGE electrophoresis was performed, followed by protein transfer onto a PVDF membrane. The membrane was subsequently blocked with 5% skimmed milk powder for 1 h prior to overnight incubation at 4 °C with primary antibodies against VEGFR-1 and vimentin (Abcam, Cambridge, UK). Following five rounds of TBST washing, secondary antibodies (1:3000, Elabscience, Wuhan, China) were added and incubated at 25 °C for 2 h. Then, blot bands were visualized with an ECL reagent (Bio-Rad, Hercules, CA, USA) and were quantified by densitometry using ImageJ 1.51j software (NIH, Bethesda, Rockville, MD, USA). The results were normalized using β-actin (Abcam, Cambridge, UK) as an internal control [42].

### 4.8. Statistical Analysis

All the data were obtained in three independent replicates, and analyzed with Graphpad Prism 6 software (Graphpad Software, San Diego, CA, USA) and represented as mean ± standard deviation. A *p*-value less than 0.05 was deemed to have statistical significance.

## 5. Conclusions

In summary, four meroterpenoids were isolated from a marine sponge *Hyrtios* sp., including two new compounds hyrtamide A (**1**) and hyrfarnediol A (**2**), and two known ones, 3-farnesyl-4-hydroxybenzoic acid methyl ester (**3**) and dictyoceratin C (**4**). Compounds **2**–**4** exhibited weak cytotoxic activities. Compounds **3** and **4** significantly inhibited the invasion of HCT-116 cells and notably suppressed the expression of VEGFR-1 and vimentin (a biomarker of EMT), suggesting that they may inhibit tumor cell metastasis by preventing the EMT process through downregulation of VEGFR-1 expression. The mechanisms behind the anti-metastatic effects of compounds **3** and **4** require further investigation.

## Figures and Tables

**Table 1:** ^1^H and ^13^C NMR data of compounds **1** and **2** in CDCl_3_.

Position	1 (600 and 150 MHz)		Position	2 (400 and 100 MHz)	
	*δ*_C_, Type	*δ*_H_, Mult. (*J* in Hz)		*δ*_C_, Type	*δ*_H_, Mult. (*J* in Hz)
1	175.3, C		1	122.3, C	
2	139.7, C		2	124.0, CH	7.44, br s
3	25.8, CH_2_	2.33, t (7.0)	3	127.3, C	
4	26.1, CH_2_	2.26, q (7.0)	4	147.0, C	
5	123.5, CH	5.15, t (7.0)	5	146.7, C	
6	136.3, C		6	114.7, CH	7.46, br s
7	39.9, CH_2_	1.98, m	1′	29.6, CH_2_	3.40, d, (6.4)
8	27.0, CH_2_	2.06, m	2′	121.3, CH	5.34, m
9	124.4, CH	5.11, m	3′	139.3, C	
10	135.2, C		4′	39.9, CH_2_	2.11, m; 1.97, m
11	39.9, CH_2_	1.98, m	5′	26.9, CH_2_	2.04, m; 1.97, m
12	26.9, CH_2_	2.06, m	6′	123.8, CH	5.10, m
13	124.4, CH	5.11, m	7′	135.9, C	
14	135.1, C		8′	39.9, CH_2_	2.11, m; 1.97, m
15	39.9, CH_2_	1.98, m	9′	26.6, CH_2_	2.11, m
16	26.9, CH_2_	2.06, m	10′	124.5, CH	5.10, m
17	124.6, CH	5.11, m	11′	131.6, C	
18	131.5, C		12′	25.9, CH_3_	1.68, s
19	25.9, CH_3_	1.68, s	13′	16.5, CH_3_	1.79, s
20	137.7, CH	6.73, q (1.5)	14′	16.3, CH_3_	1.60, s
21	46.6, CH_2_	3.91, t (1.5)	15′	17.9, CH_3_	1.60, s
22	16.3, CH_3_	1.60, s	1-C=O	167.4, C	
23	16.2, CH_3_	1.60, s	-OCH_3_	52.2, CH_3_	3.87, s
24	16.2, CH_3_	1.60, s			
25	17.9, CH_3_	1.60, s			

**Table 2:** Cytotoxic activities of compounds **2**–**4** against HCT-116.

Compound	IC_50_ (μM)
**2**	41.6 ± 3.8
**3**	45.0 ± 3.0
**4**	37.3 ± 3.3
Doxorubicin	3.8 ± 0.1

## Data Availability

The data presented in this study can be accessed in the Supplementary Materials; further inquiries can be directed to the corresponding author.

## Supplementary Materials

The following supporting information can be downloaded at: https://www.mdpi.com/article/10.3390/md22040183/s1, Figures S1–S16: HRESIMS, UV, ^1^H and ^13^C NMR, HSQC, HMBC, COSY, and ROESY spectra of hyrtamide A (**1**) and hyrfarnediol A (**2**). Figures S17–S22: ESI-MS, ^1^H and ^13^C NMR spectra of 3-farnesyl-4-hydroxybenzoic acid methyl ester (**3**) and dictyoceratin C (**4**).
