# Supplementary material for: Meroterpenoids from Marine Sponge Hyrtios sp. and Their Anticancer Activity against Human Colorectal Cancer Cells
Source: Mar Drugs. 2024 Apr 19;22(4):183. doi: 10.3390/md22040183 (PMC11051118; doi:10.3390/md22040183)
Supplement: Supplementary file 1 [file marinedrugs-22-00183-s001.zip › marinedrugs-2978340-supplementary.pdf]

Supporting Information

Meroterpenoids from Marine Sponge *Hyrtios* sp. and Their Anticancer Activity against Human Colorectal Cancer Cells

Jie Wang [1,†,*], Yue-Lu Yan [1,†], Xin-Yi Yu [1], Jia-Yan Pan [1], Xin-Lian Liu [1], Li-Li Hong [2,*] and Bin Wang [1,*]

[1] Zhejiang Provincial Engineering Technology Research Center of Marine Biomedical Products, School of Food and Pharmacy, Zhejiang Ocean University, Zhoushan 316022, China; yyuelu03@163.com (Y.-L.Y.); 17355410304@163.com (X.-Y.Y.); pjy2388@163.com (J.-Y.P.); lxl19960817@163.com (X.-L.L.)

[2] Research Center for Marine Drugs, Department of Pharmacy, Ren Ji Hospital, School of Medicine, Shanghai Jiao Tong University, Shanghai 200127, China.

* Correspondence: 011103@zjou.edu.cn (J.W.); hongll0792@sjtu.edu.cn (L.-L.H.); wangbin@zjou.edu.cn (B.W.);

† These authors contributed equally to this work.

List of Content

Figure S1. HRESIMS spectrum of compound **1**

Figure S2. UV Spectrum of compound **1**

Figure S3. ^{1}H NMR (600 MHz) Spectrum of compound **1** in $CDCl_3$

Figure S4. ^{13}C NMR (150 MHz) Spectrum of compound **1** in $CDCl_3$

Figure S5. HMQC Spectrum of compound **1** in $CDCl_3$

Figure S6. HMBC Spectrum of compound **1** in $CDCl_3$

Figure S7. COSY Spectrum of compound **1** in $CDCl_3$

Figure S8. NOESY Spectrum of compound **1** in $CDCl_3$

Figure S9. HRESIMS spectrum of compound **2**

Figure S10. UV Spectrum of compound **2**

Figure S11. ^{1}H NMR (400 MHz) Spectrum of compound **2** in $CDCl_3$

Figure S12. ^{13}C NMR (100 MHz) Spectrum of compound **2** in $CDCl_3$

Figure S13. HMQC Spectrum of compound **2** in $CDCl_3$

Figure S14. HMBC Spectrum of compound **2** in $CDCl_3$

Figure S15. COSY Spectrum of compound **2** in $CDCl_3$

Figure S16. NOESY Spectrum of compound **2** in $CDCl_3$

Figure S17. ESI-MS spectrum of compound **3**

Figure S18. ^{1}H NMR (600 MHz) Spectrum of compound **3** in $CDCl_3$

Figure S19. ^{13}C NMR (150 MHz) Spectrum of compound **3** in $CDCl_3$

Figure S20. ESI-MS spectrum of compound **4**

Figure S21. ^{1}H NMR (600 MHz) Spectrum of compound **4** in $CDCl_3$

Figure S22. ^{13}C NMR (150 MHz) Spectrum of compound **4** in $CDCl_3$

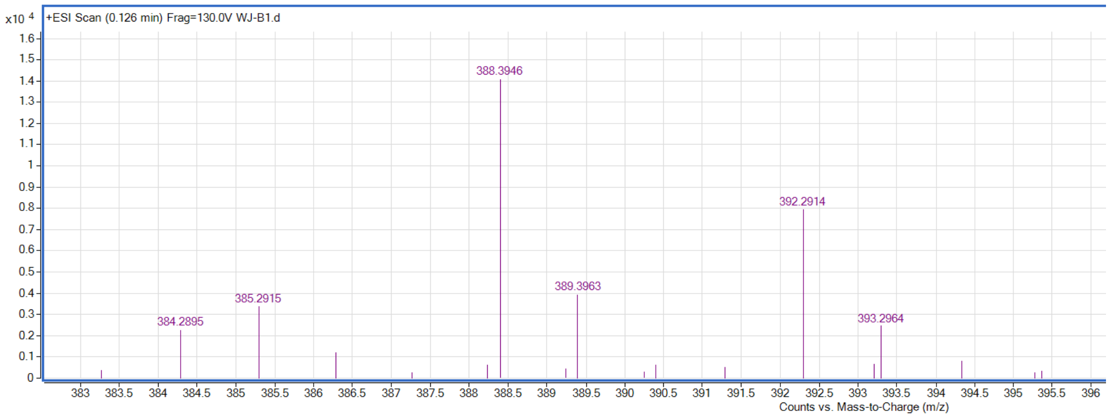

Figure S1. HRESIMS spectrum of compound **1**

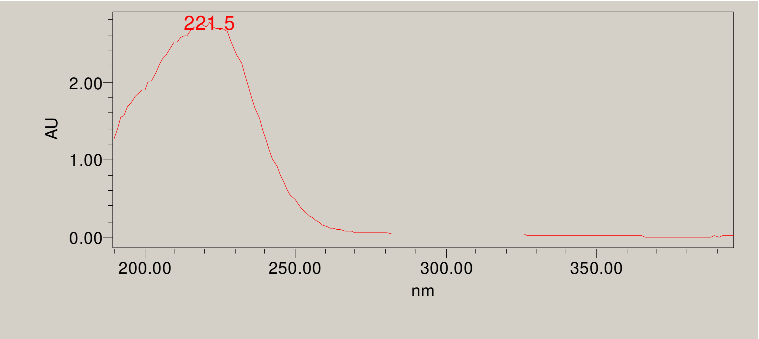

Figure S2. UV Spectrum of compound **1**

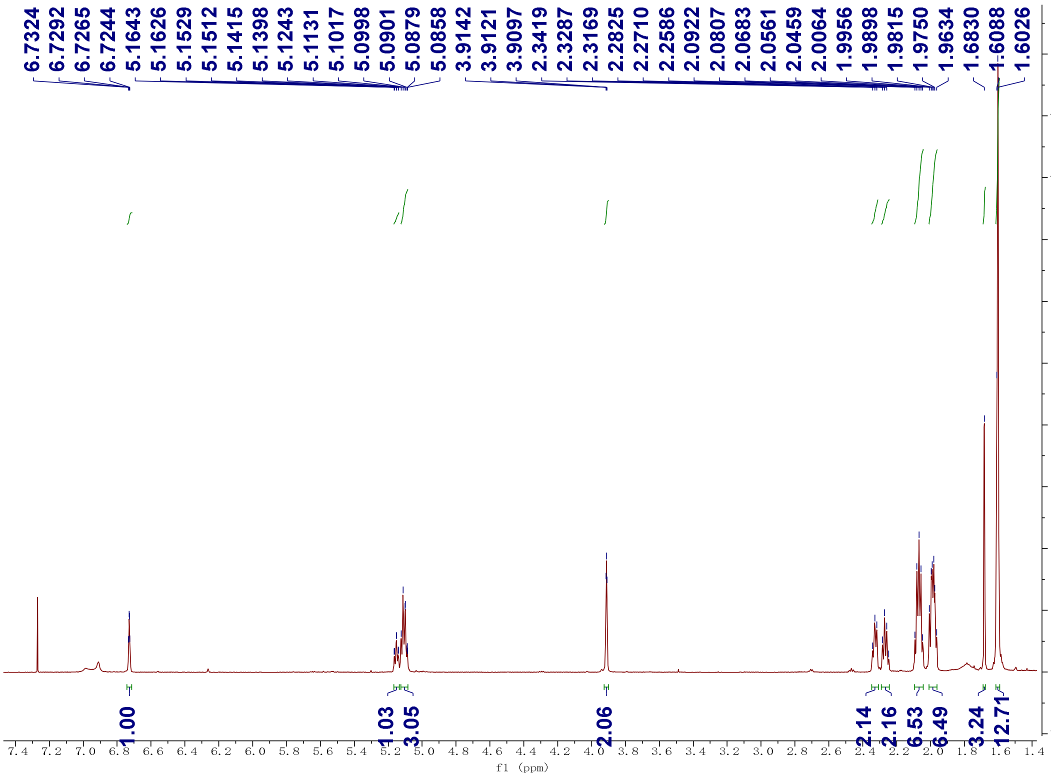

Figure S3. ^{1}H NMR (600 MHz) Spectrum of compound **1** in $CDCl_3$

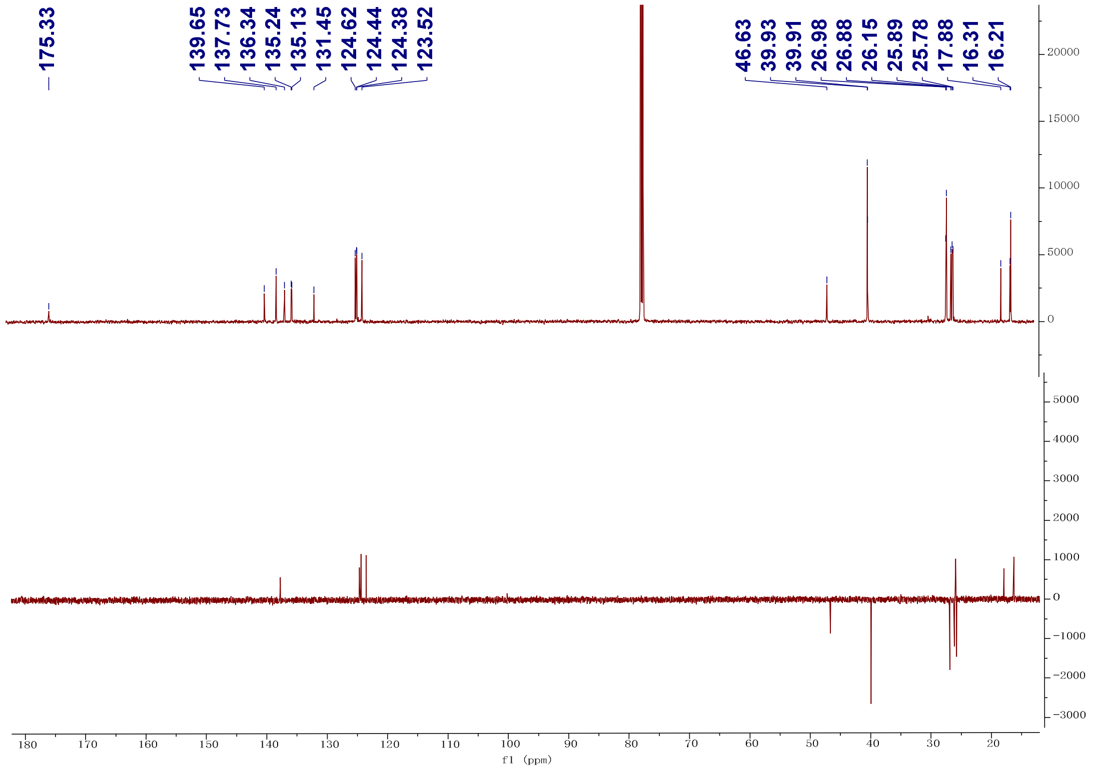

Figure S4. ^{13}C NMR (150 MHz) Spectrum of compound **1** in $CDCl_3$

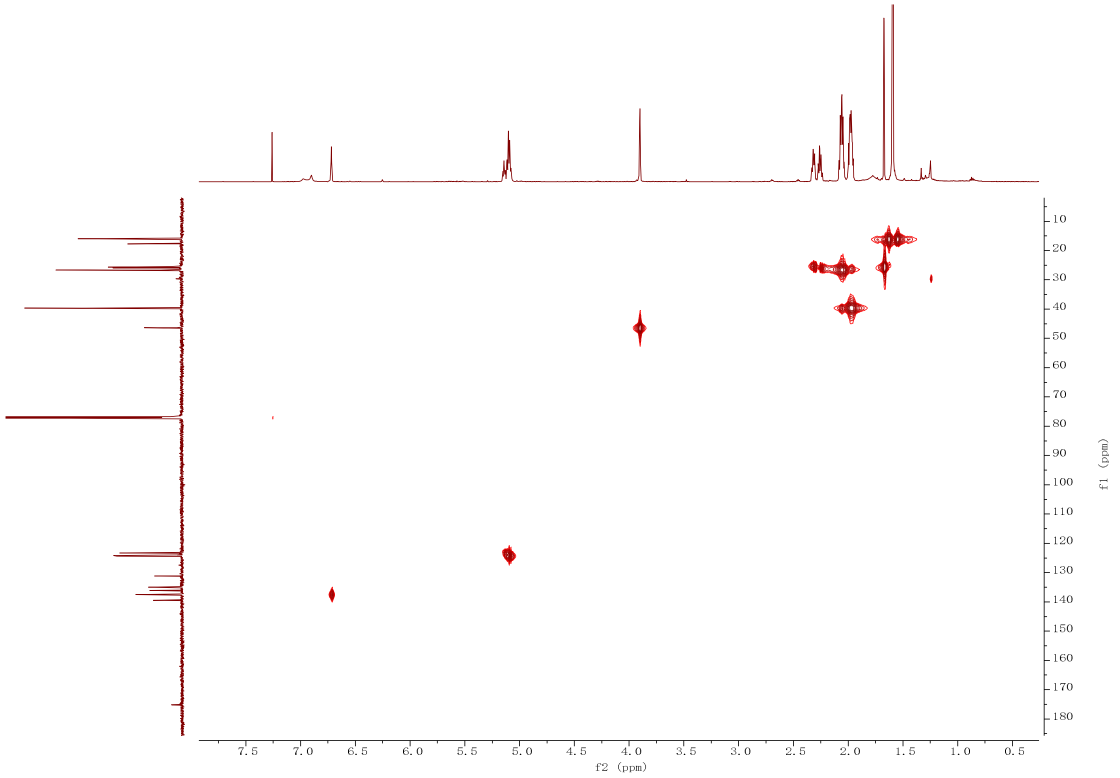

Figure S5. HMQC Spectrum of compound **1** in $CDCl_3$

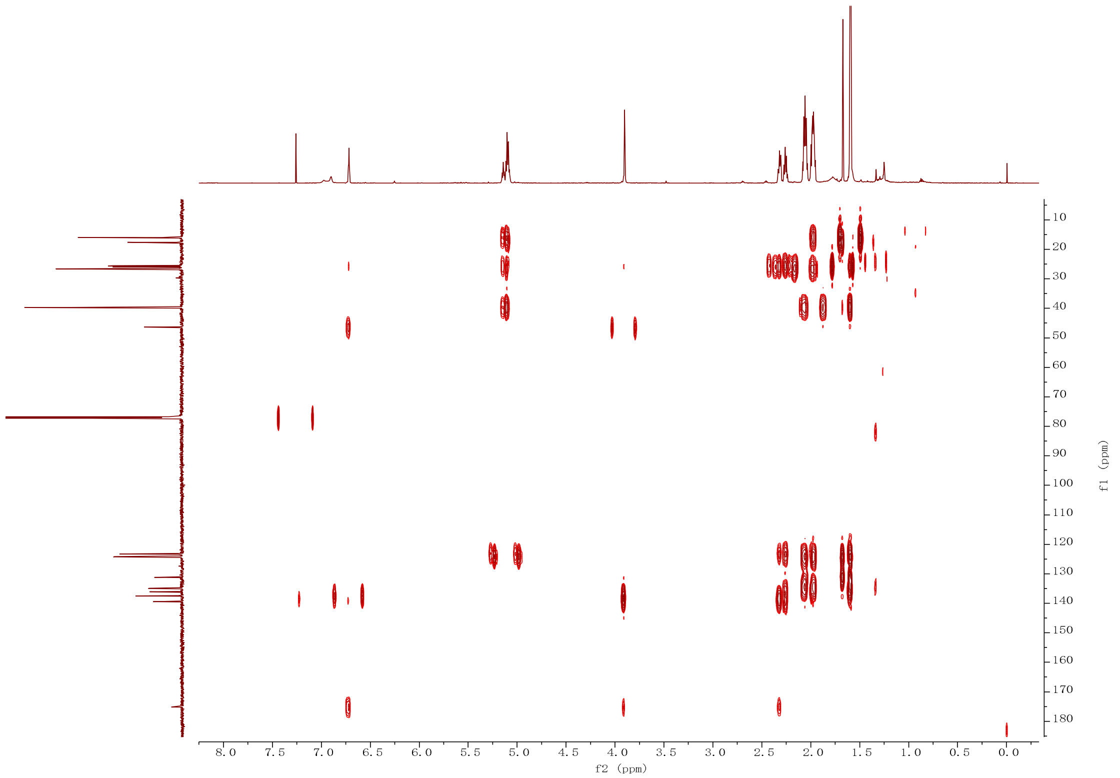

Figure S6. HMBC Spectrum of compound **1** in $CDCl_3$

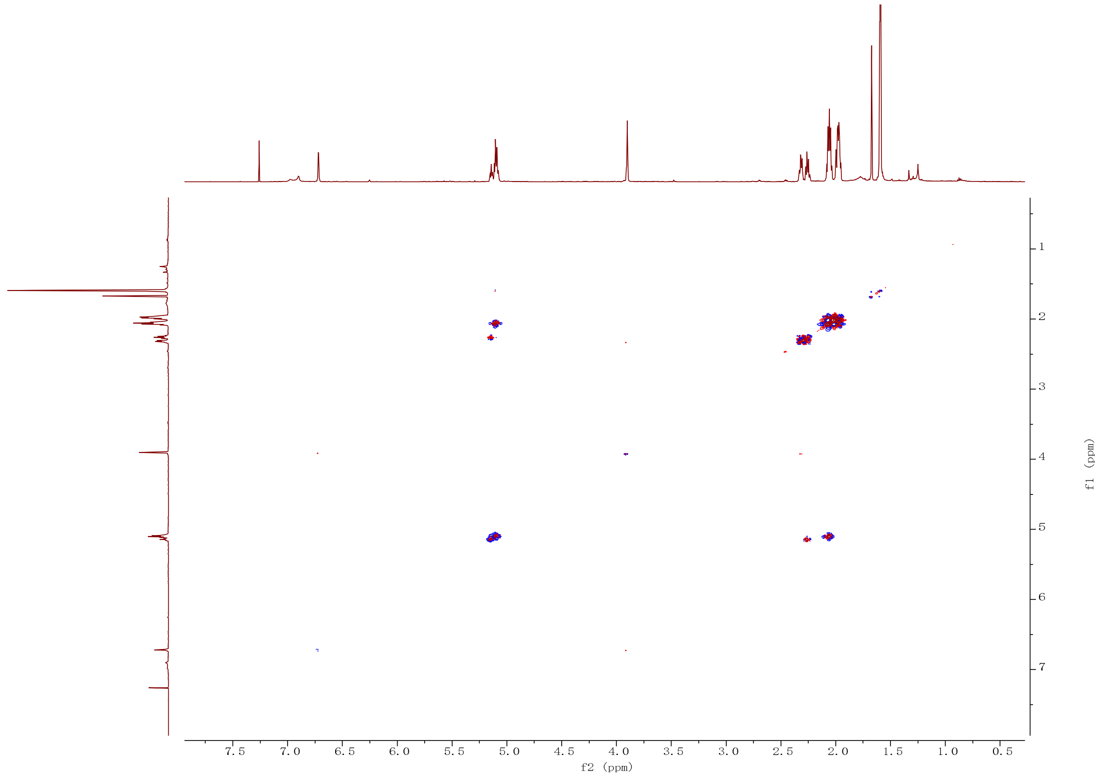

Figure S7. COSY Spectrum of compound **1** in $CDCl_3$

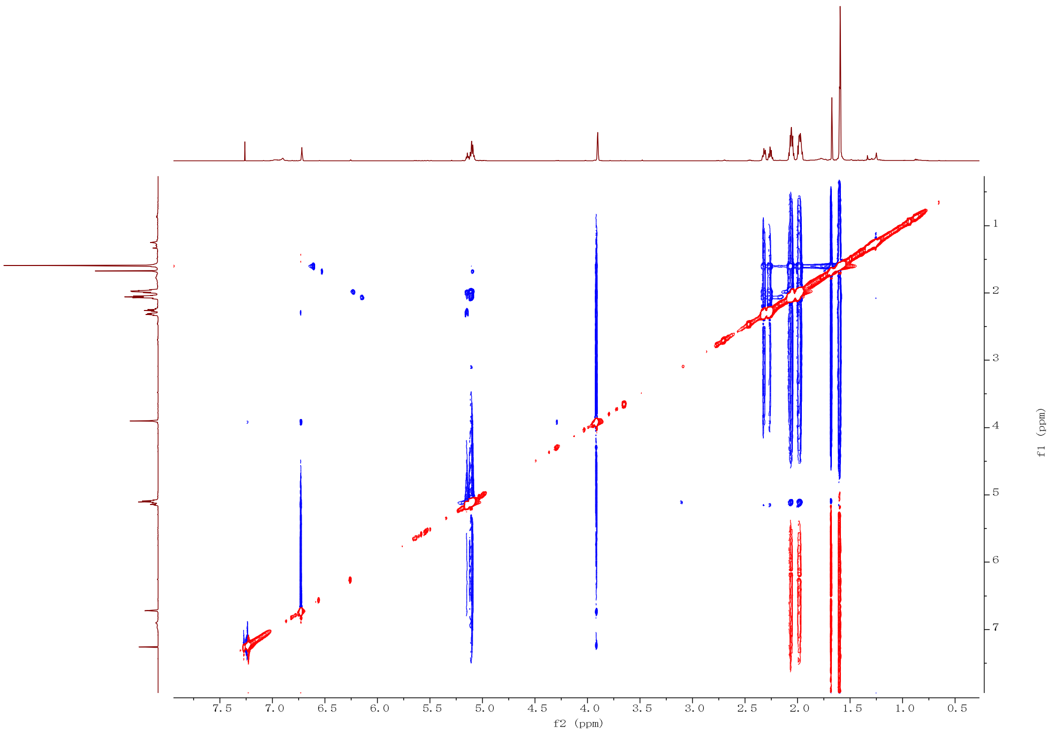

Figure S8. NOESY Spectrum of compound **1** in $CDCl_3$

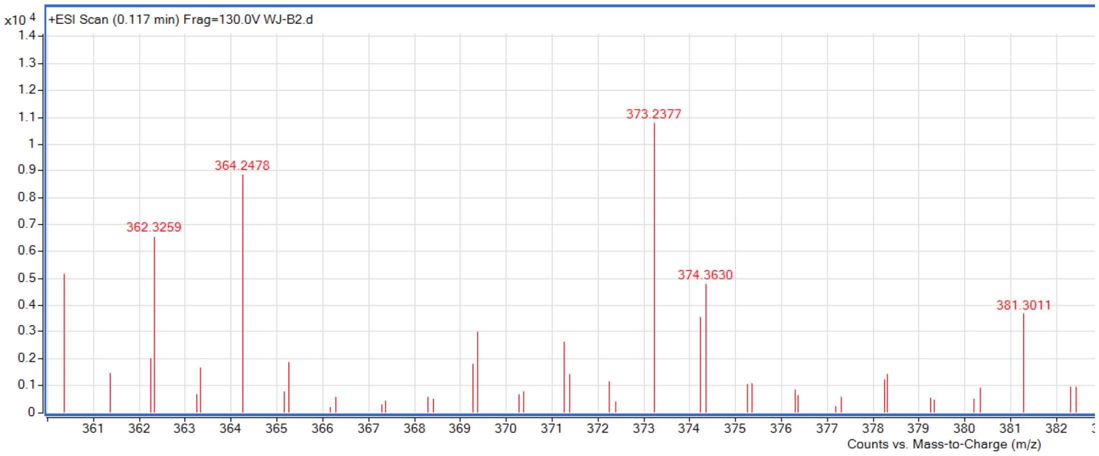

Figure S9. HRESIMS spectrum of compound **2**

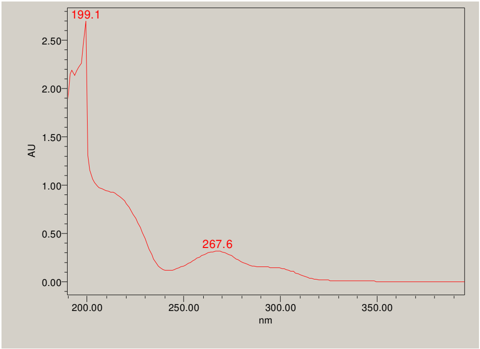

Figure S10. UV Spectrum of compound **2**

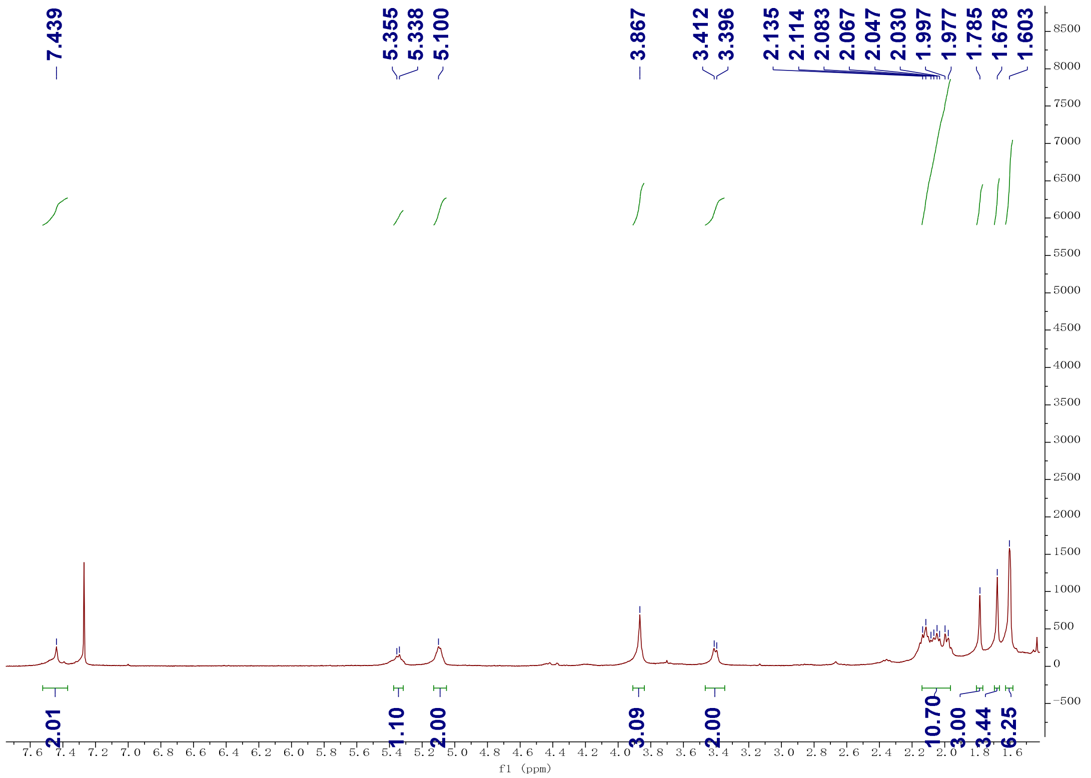

Figure S11. ^{1}H NMR (400 MHz) Spectrum of compound **2** in $CDCl_3$

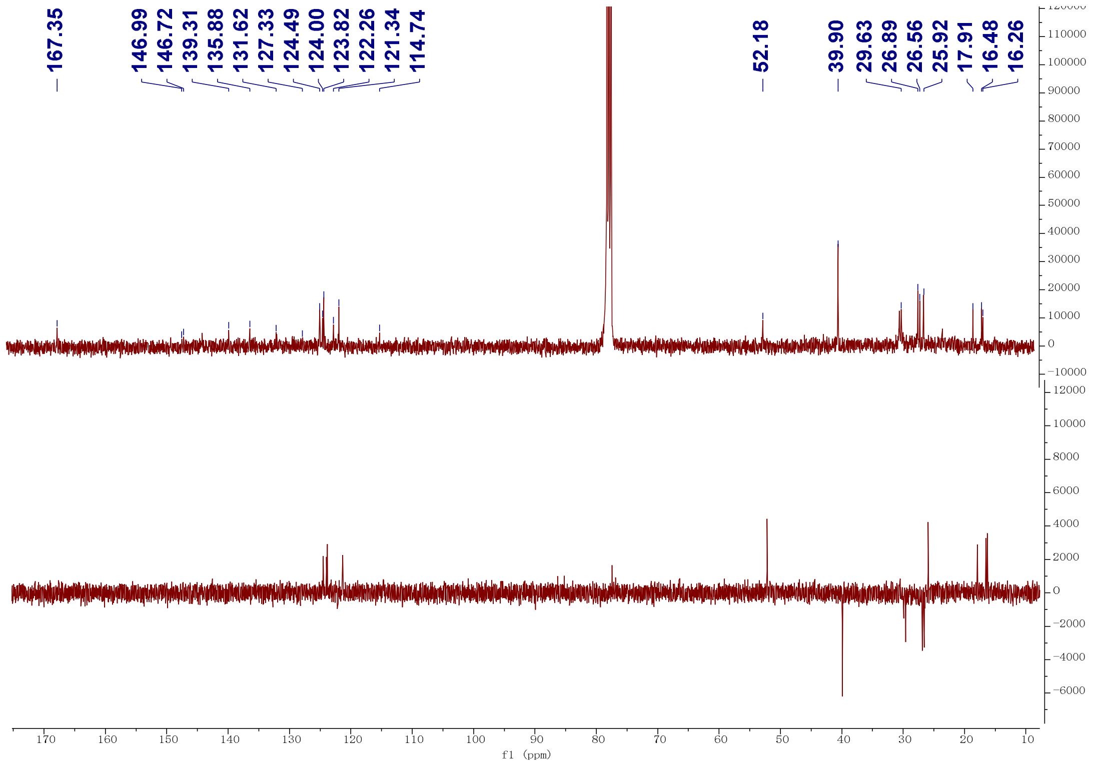

Figure S12. ^{13}C NMR (100 MHz) Spectrum of compound **2** in $CDCl_3$

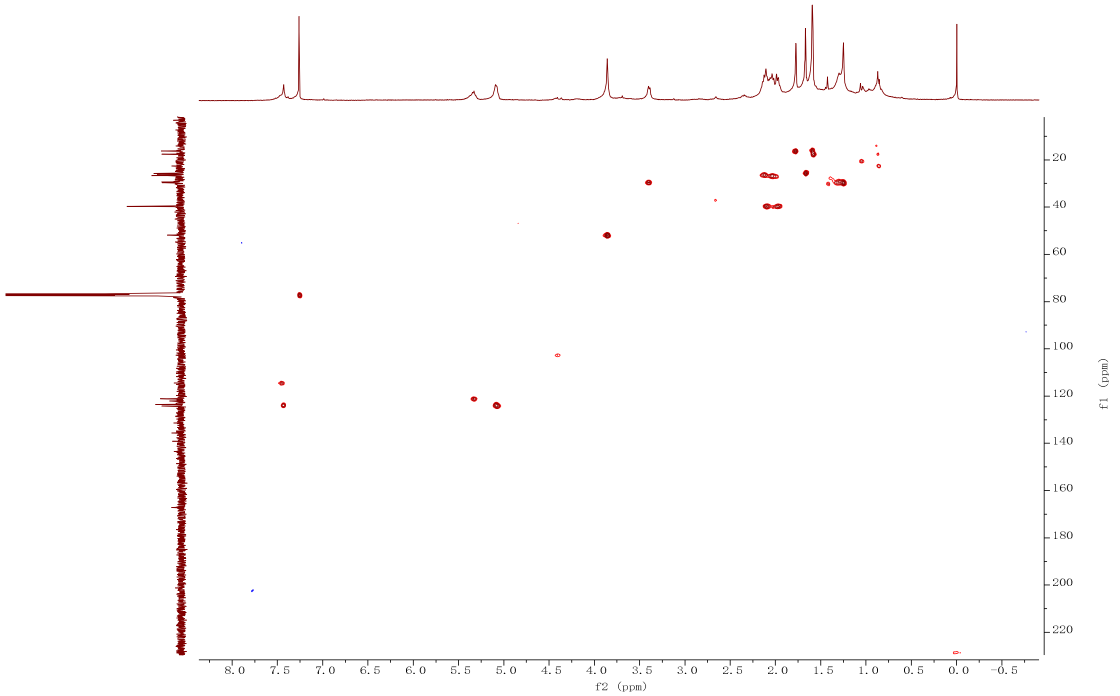

Figure S13. HMQC Spectrum of compound **2** in $CDCl_3$

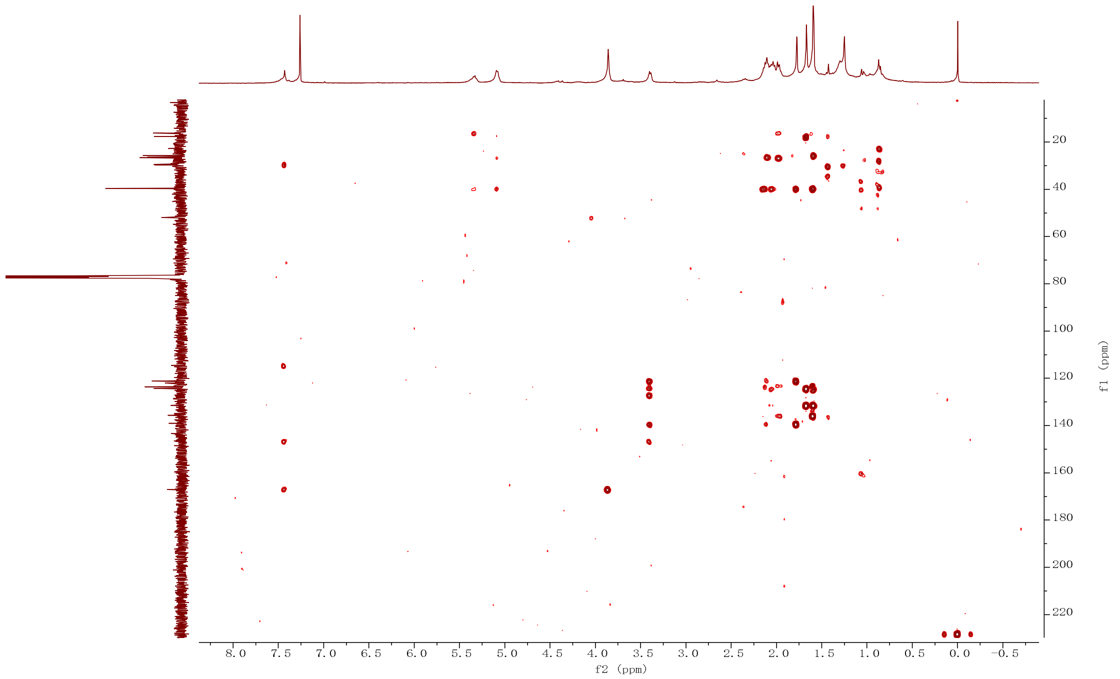

Figure S14. HMBC Spectrum of compound **2** in $CDCl_3$

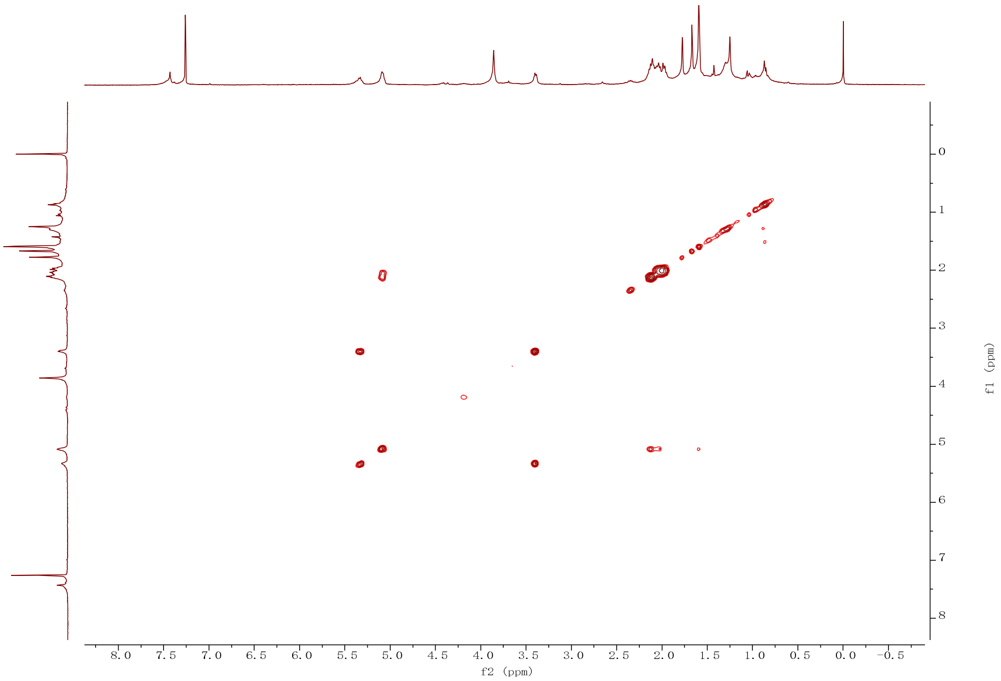

Figure S15. COSY Spectrum of compound **2** in $CDCl_3$

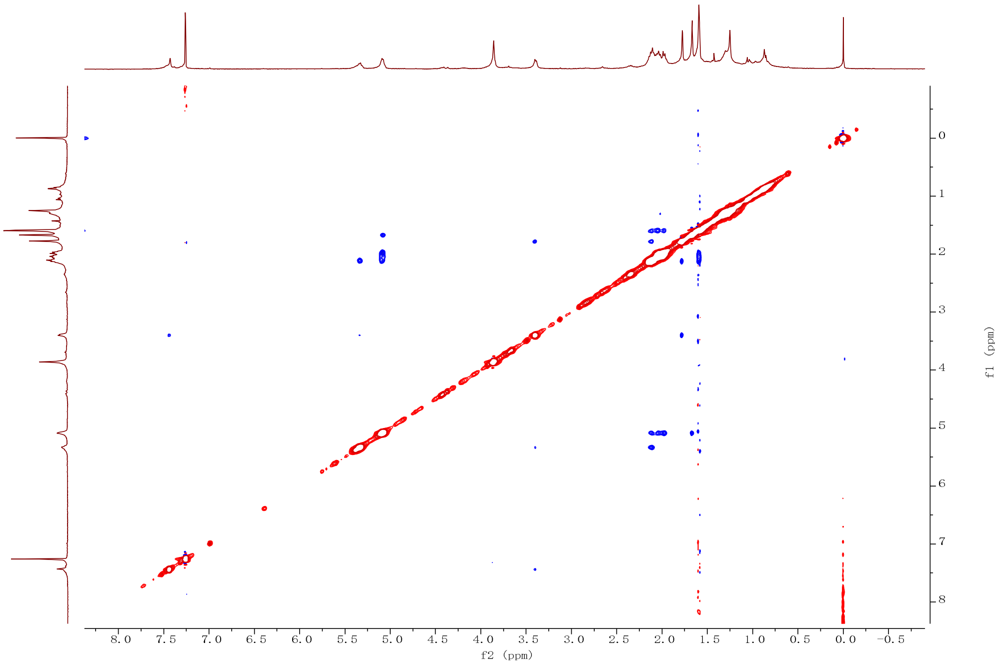

Figure S16. NOESY Spectrum of compound **2** in $CDCl_3$

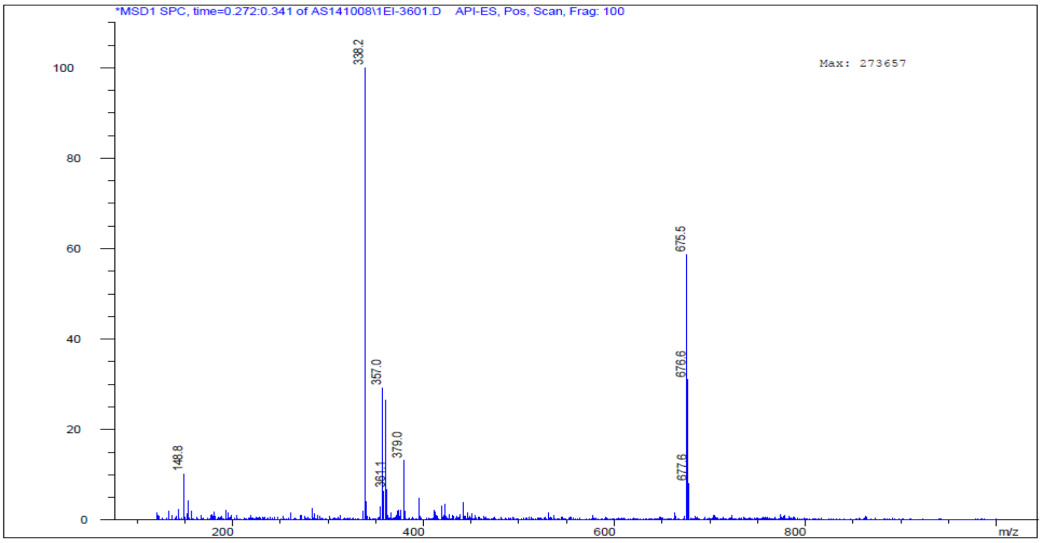

Figure S17. ESI-MS spectrum of compound **3**

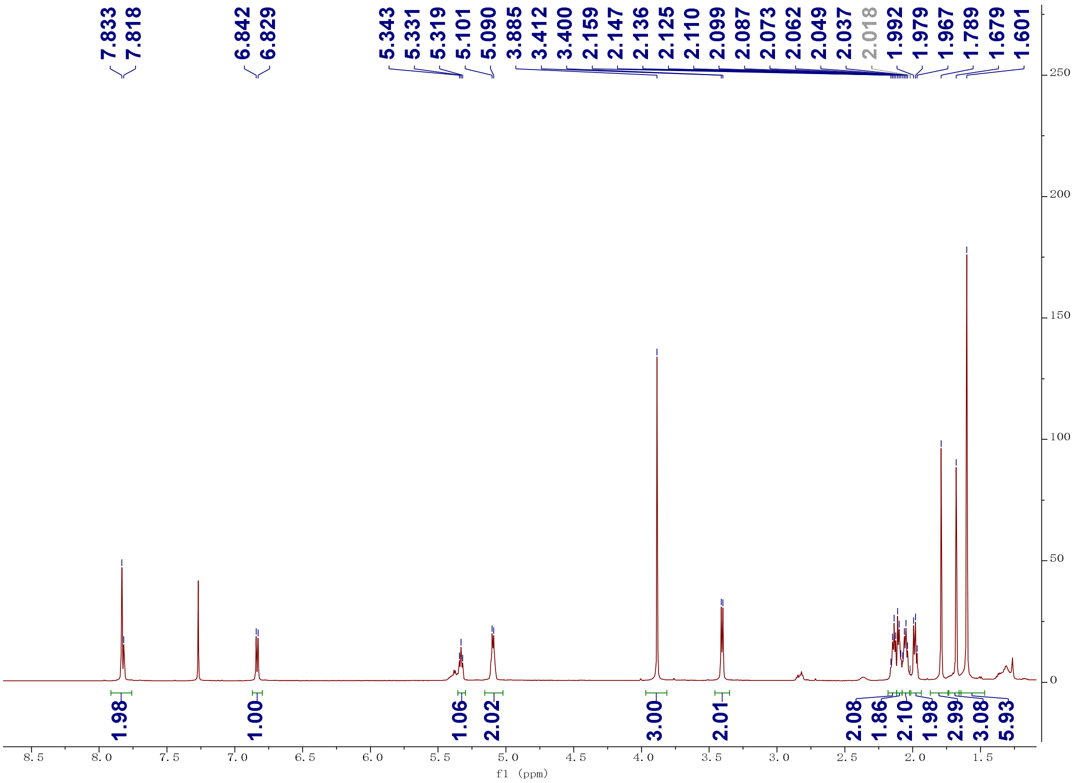

Figure S18. ^{1}H NMR (600 MHz) Spectrum of compound **3** in $CDCl_3$

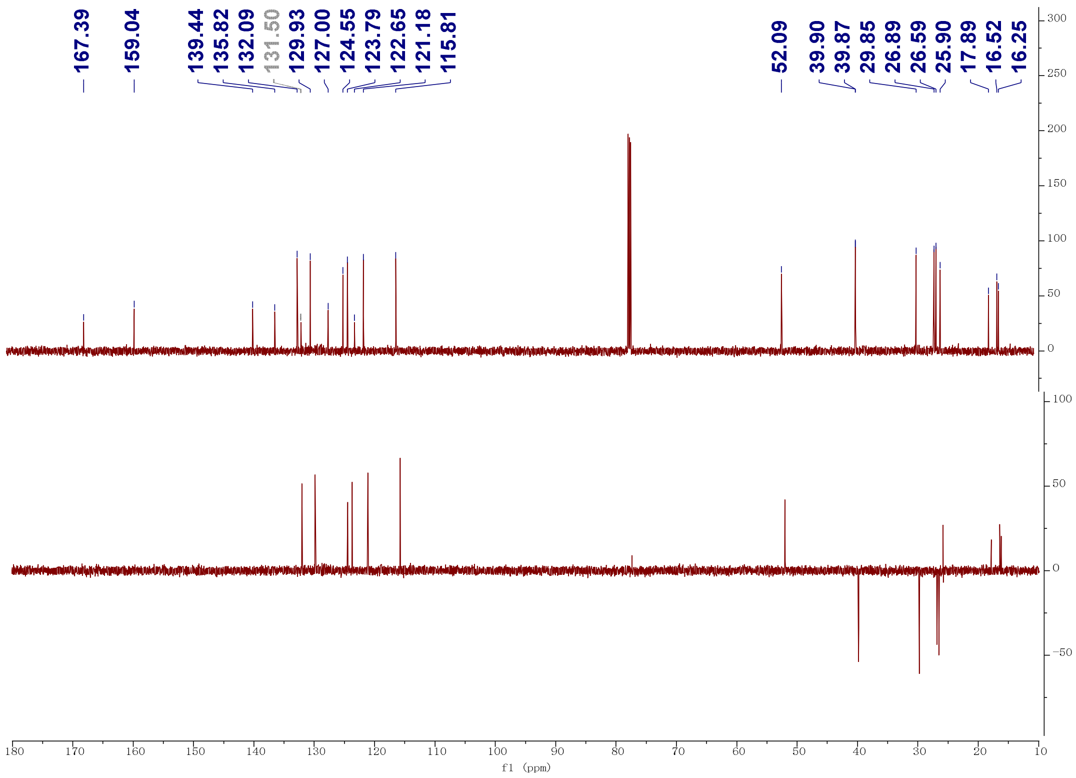

Figure S19. ^{13}C NMR (150 MHz) Spectrum of compound **3** in $CDCl_3$

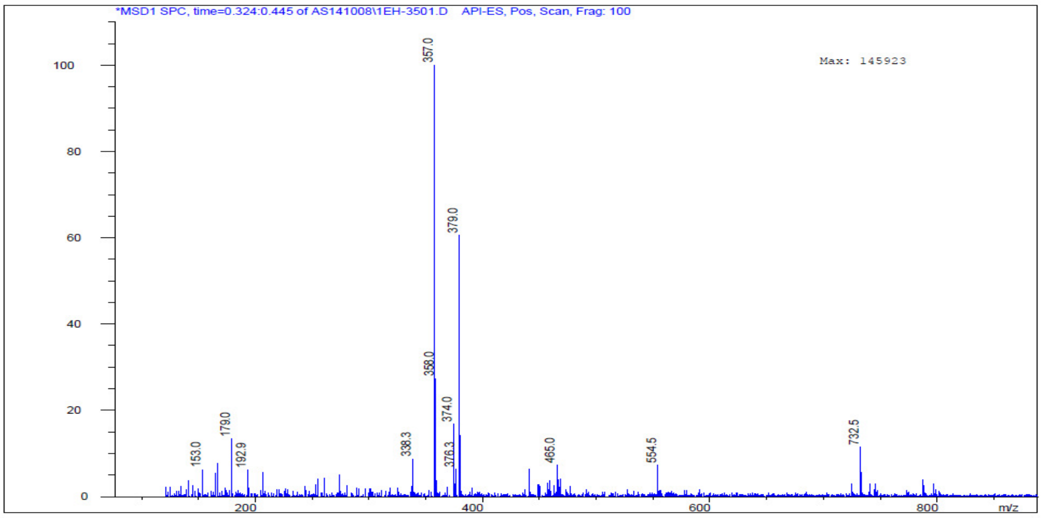

Figure S20. ESI-MS spectrum of compound **4**

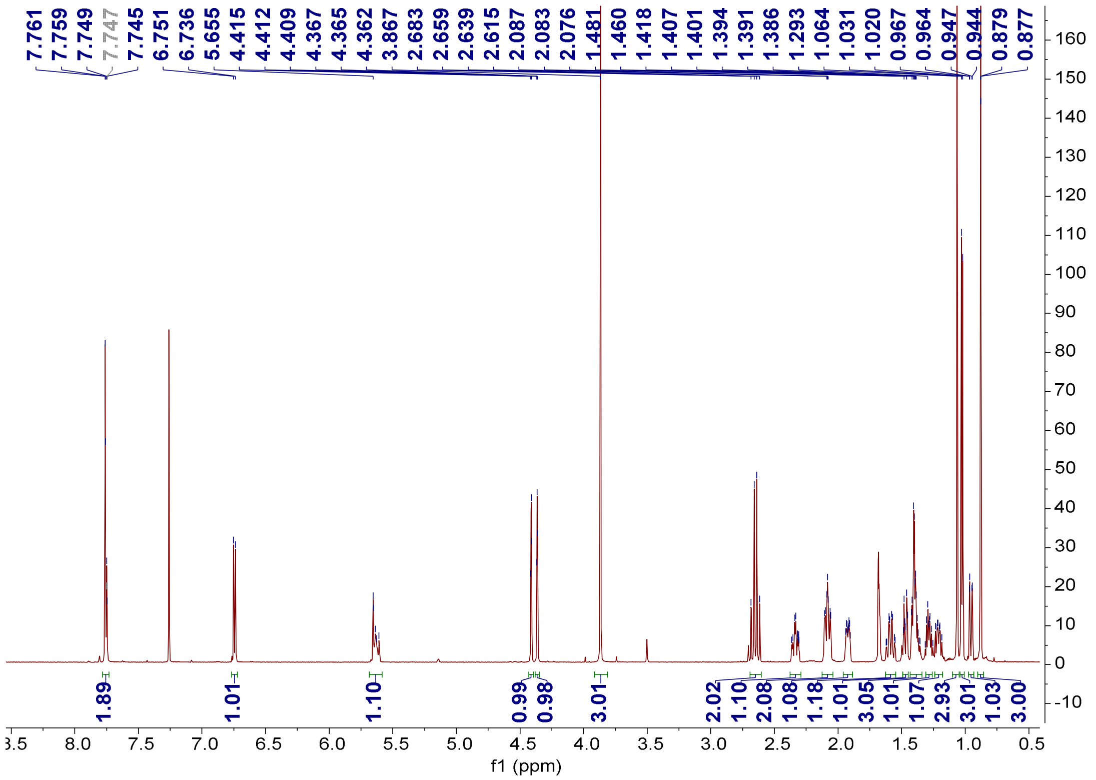

Figure S21. ^{1}H NMR (600 MHz) Spectrum of compound **4** in $CDCl_3$

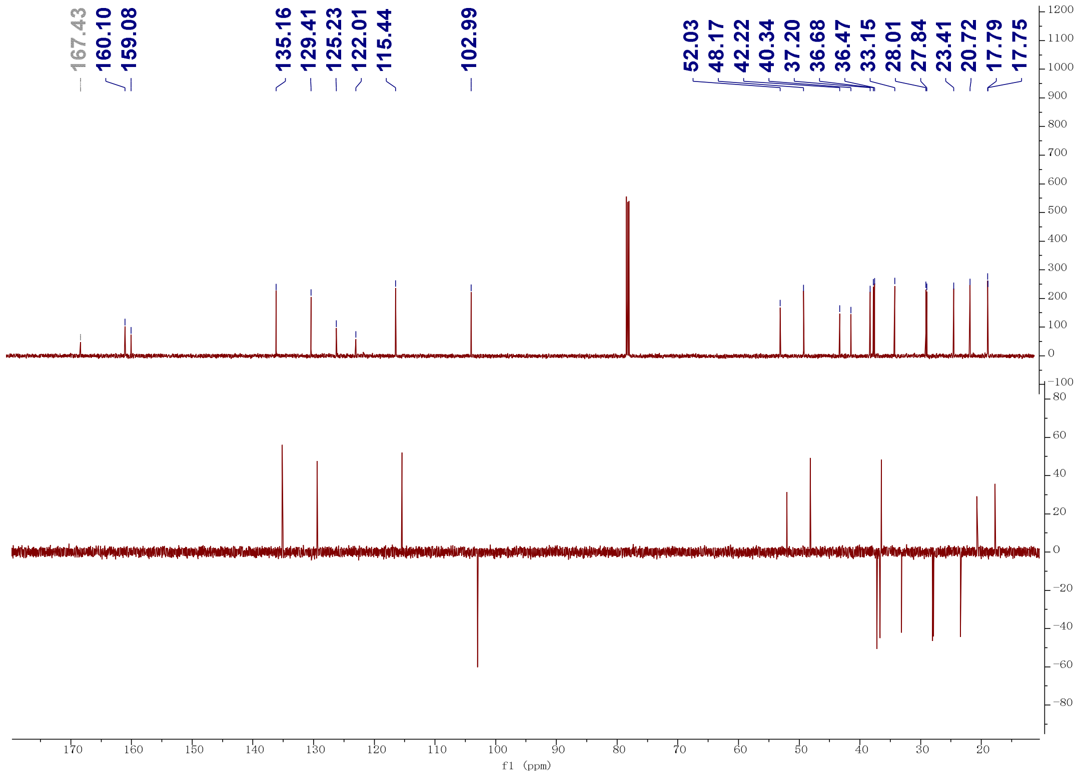

Figure S22. ^{13}C NMR (150 MHz) Spectrum of compound **4** in $CDCl_3$
